# Lithium Ion Sensors

**DOI:** 10.3390/s17102430

**Published:** 2017-10-24

**Authors:** Megi Kamenica, Raghuram Reddy Kothur, Alison Willows, Bhavik Anil Patel, Peter J. Cragg

**Affiliations:** School of Pharmacy & Biomolecular Sciences, University of Brighton, Huxley Building, Brighton BN2 4GJ, UK; M.Kamenica1@uni.brighton.ac.uk (M.K.); raghuramreddy935@gmail.com (R.R.K.); A.D.Willows@brighton.ac.uk (A.W.); B.A.Patel@brighton.ac.uk (B.A.P.)

**Keywords:** lithium, ionophore, sensor, colorimetry, fluorescence, electrochemical detection

## Abstract

The detection and monitoring of lithium in environmental and clinical settings is becoming increasingly important. In this review, sensors incorporating conductive polymers and lithium bronzes are discussed, together with electrochemical and spectroscopic approaches. Ionophore-based methods have been employed extensively, with varying degrees of selectivity and sensitivity, and these are discussed in depth.

## 1. Introduction

Lithium is present in the Earth’s crustal rocks at a concentration of 20–60 ppm, and in 0.2 ppm in seawater, from which it is extracted for a variety of uses, including lightweight metal alloys, batteries, ceramics and glasses, and medication [1]. Consequently, it is ubiquitous but present in very low concentrations except where geological conditions have resulted in localised increases of lithium-rich minerals such as petalite, spodumene and lepidolite. Much of the lithium extracted today comes from lithium-rich brine pools in countries adjoining the Andes in South America. 

## 2. The Need to Detect Li^+^ in Aqueous Solutions

Lithium salts, notably lithium carbonate, have been widely and effectively used in the treatment of bipolar disorders for more than 50 years, and have also been applied to patients diagnosed with dementia-related conditions [2]. It is critical to determine and monitor the correct dose for every patient, as the levels of lithium are affected by each individual’s metabolism and other variables. The ability to measure Li^+^ concentrations in biofluids such as blood and urine accurately and precisely is consequently a vital target for analysis. Uncontrolled high concentrations of Li^+^ can permanently affect the nervous system and kidneys [3]. The beneficial effects of Li^+^ salts are directly associated with the amount of the medicine taken, but as the pharmacokinetic profile of each person is different due to intrinsic factors, the concentration must be checked continuously to minimise any risk of harm to the body. Ideally, lithium detection should be done through measurements in blood serum so that interventions in response to variations in Li^+^ concentration can be made rapidly [4]. One key issue is the ability to measure therapeutic levels of Li^+^, which range from 5 × 10^−4^ mol dm^−3^ to 1.5 × 10^−3^ mol dm^−3^, in the presence of Na^+^ at physiological levels in the region of 0.15 mol dm^−3^. 

The expanding use of lithium ion batteries, in particular, is likely to bring more environmental exposure through leaching of landfill. Since they have a high particular energy and density, lithium ion batteries are used preferentially where small, long-lived batteries are required, such as medical implants or mobile phones; however there has been little attention paid to the eventual fate of the metal.

Regardless of the source, metal contamination in water is a critical issue in light of the potential risks to human wellbeing and the wider environment. This problematic topic has prompted firm guidelines on the concentrations of metal cations allowed to be present in water [5]. Free metal ion concentrations in solution, in most cases, eventually define the bioavailability and harmfulness of these metals [6]. Several commercial instruments with the capacity to detect lithium exist, starting in 1987 with NOVA Biomedical’s ion selective electrode; however, most of the existing analytical systems can be expensive and the samples require pre-treatment prior to analysis. In the case of lithium, the coupling of electrochemical techniques to macrocyclic molecules can establish high sensitivity, reproducibility, low detection limits, and high selectivity due to complementarity between the cation’s electrostatic properties and those of the macrocyclic cavity. Portable devices can be devised to take advantage of these desirable characteristics, which will allow accurate detection of lithium in situ, whether in hospitals in remote locations, landfill sites or offshore monitoring. 

## 3. Sensors

### 3.1. Emission-Based Sensors

Different methods have been used to determine serum concentrations of lithium salts, including atomic emission spectrophotometry (AES) and ion-selective electrodes (ISE). The most promising are electrochemical sensors which offer accurate examination in situ [7,8]. These devices are small, provide rapid results, and are low in price; however, the simplest method to detect lithium is from its characteristic red flame colour with an emission at 670 nm. In 1964 Riley and Tongudai analysed 30 samples of seawater from different oceans, to determine Li^+^ contents by flame spectrophotometry [9]. It was found that Li^+^ was proportional to the concentration of chloride in water but it was also noted that the presence of high concentrations of cations and anions in sea water prevented the direct determination of Li^+^, and pre-treatment using ion exchange chromatography was necessary to separate the ions.

Clinical methods to monitor Li^+^ employ either flame emission photometry (FEP) or atomic absorbance spectroscopy (AAS) [7]; however such instrumentation is not available in most hospital laboratories, and therefore frequent monitoring is not possible. These approaches also are time consuming due to excessive sample preparation, and do not offer high throughput or near-patient monitoring capability. The use of simple electrochemical devices, such as the blood glucose monitors used by diabetics, provides the solution. Thus, an essential objective would be to develop a device based on electrochemical Li^+^ sensors. 

Another proposed analysis for Li^+^ is X-ray fluorescence spectrometry (XRF). However, this method is not designed to detect the lighter elements, and as the fluorescence yield of lithium is very small, it remains of limited applicability. In 2011 Zawiska and Sitko studied the Li^+^ concentration in bottled water to determine if they were appropriate for drinking [10]. Because of the low concentrations of Li^+^, XRF was used, by measurements of iron radiation after stoichiometric precipitation of the lithium as potassium lithium periodatoferrate(III). The limit of detection was determined to be 1 μg Li, although the lowest concentration measured was 20 μg. The advantages of the method were its simplicity, speed, and low-cost. The determination of Li^+^ as KLiFeIO_6_ was possible because the complex forms stoichiometrically even in the presence of excess (50:1) iron.

### 3.2. Conductive Polymer Sensors

**Scheme 1:**
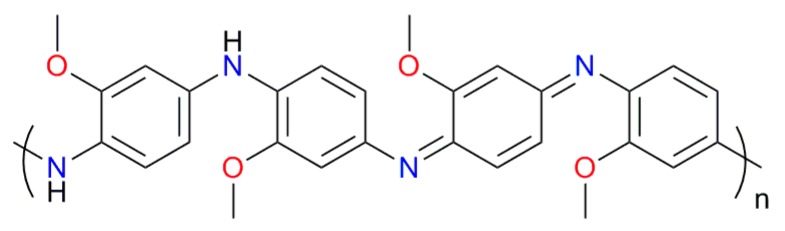
Poly(*o*-methoxyaniline) (**1**).

A study by Lindino in 2011 demonstrated that poly(*o*-methoxyaniline) (**1**) (Scheme 1) can be used to measure Li^+^ ions in solution over a concentration range from 1.0 × 10^−5^ to 1.0 × 10^−4^ mol dm^−3^ [11]. The data show that the addition of Li^+^ to the assembly influenced the electrochemical effects of poly(*o*-methoxyaniline) thus providing a detection capability. To reduce the contact time between the polymer and lithium ions, the study suggested that the process of flow injection could be optimised.

### 3.3. Lithium Bronze Electrodes

Solid state electrodes based on lithium vanadium bronze (Li_x_V_2_O_5_) and lithium molybdenum bronze (Li_x_MoO_3_) have been investigated by Gadzekpo et al.; however, Li_x_V_2_O_5_ failed to respond to Li^+^ until it was powdered and incorporated into a PVC (polyvinyl chloride) matrix [12]. Li_x_MoO_3_ gave a near-Nernstian response in crystalline form, but salts caused interference.

### 3.4. Ionophore-Based Sensors

**Scheme 2:**
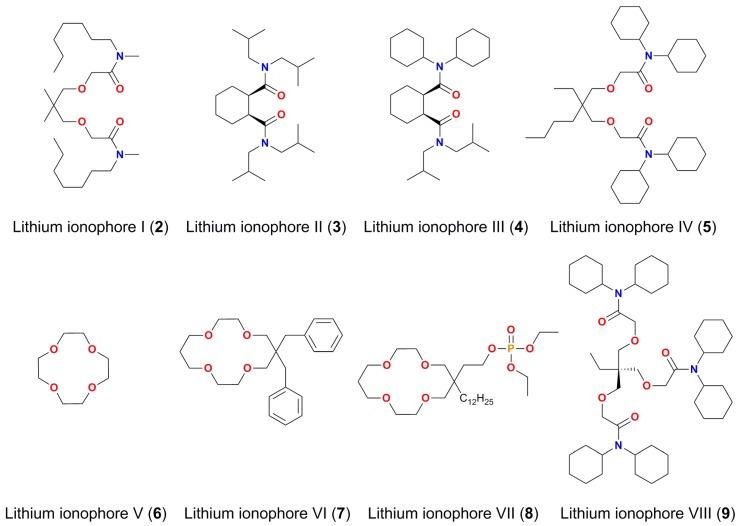
Lithium ionophores I through VIII: *N*,*N*′-diheptyl-*N*,*N*′,5,5-tetramethyl-3,7-dioxanonanediamide (**2**) [15], *N*,*N*,*N*′,*N*′-tetraisobutyl-*cis*-cyclohexane-1,2-dicarboxamide (**3**) [16], *N*,*N*-dicyclohexyl-*N*′,*N*′-diisobutyl-*cis*-cyclohexane-1,2-dicarboxamide (**4**) [17], 6,6-dibenzyl-1,4,8,11-tetraoxacyclotetradecane (**5**) [18], 12-crown-4 (**6**) [14], 6,6-dibenzyl-1,4,8,11-tetraoxacyclotetradecane (**7**) [19], 2-(6-dodecyl-1,4,8,11-tetraoxacyclotetradec-6-yl)ethyl diethyl phosphate (**8**) [20], 2-[2,2-bis[[2-(dicyclohexylamino)-2-oxoethoxy]methyl]butoxy]-*N*,*N*-dicyclohexylacetamide (**9**) [21].

In 1967 Pedersen published his groundbreaking study of the crown ethers, in which he noted a complementarity between the size of the macrocycle and the cations it bound [13]. While he reported the syntheses of benzo- and cyclohexyl-12-crown-4, 12-crown-4 itself had first been patented in 1957 [14], as Pedersen acknowledged in his paper. Along with other Li^+^-binding compounds it has joined the class of lithium ionophores as shown in Scheme 2. 

The ionophores have two applications: by virtue of their affinities for Li^+^, they can be incorporated into ion-selective membranes or electrodes and also, due to their lipophilicities, can extract Li^+^ from aqueous solution into organic solvents in preparation for concentration and spectrophotometric analyses. 

**Scheme 3:**
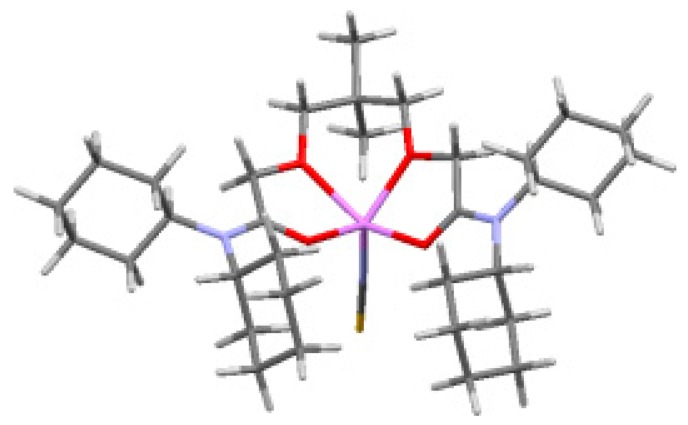
The 1:1 complex formed between **5** and lithium isothiocyanate.

In 1977 Kirsch et al. reported the first example of the selectivity behaviour of a Li^+^-targeting ionophore, **2**, both in membranes and as an extracting agent from ethanol solution [15]. Shortly afterwards, Zhukov et al. reported a ion-selective solvent polymeric membrane incorporating **3** which was 100 times more selective for Li^+^ than for other alkali metal ions and about 1000 times more selective for Li^+^ over alkaline earth metal cations [16]. Compound **4**, variation on **3**, which incorporated both cyclohexyl and isobutyl substituents was reported in a patent application by Simon, although no claims of superior selectivity were made [17]. Metzger et al. took the chelating aspects from **2** and incorporated substituents found in **4** to give compound **5**, which had a Li^+^:Na^+^ selectivity of 80:1 in lipophilic liquid membranes [18]. Response times for these ion-selective liquid membrane electrodes were improved, and lifetimes increased, which allowed measurements of Li^+^ in blood serum to be made within the clinically relevant concentration range. A complex between Li^+^ and **5** formed and was shown by X-ray crystallography to have the expected 1:1 stoichiometry (Scheme 3).

While 12-crown-4, **6**, is generally cited as a Li^+^-selective crown ether, it suffers from having a broad range of solubilities, as it is miscible with water and dissolves in most organic solvents [14]. This will compromise the stability of any membrane incorporating **6**, as well as making partitioning between aqueous and organic phases problematic. Despite these shortcomings, the importance of this compound cannot be overstated in the context of Li^+^-sensors, as the ‘fit’ between **6** and Li^+^ has been instrumental in the design of other macrocyclic chelating agents, such as **7** and **8**. 

**Scheme 4:**
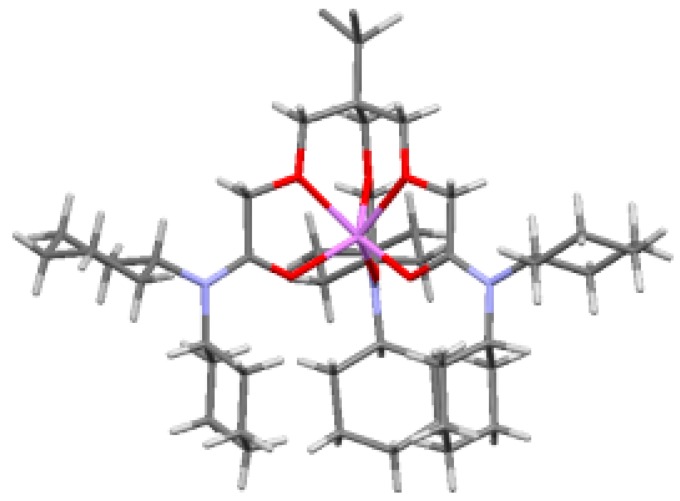
The 1:1 complex formed between **9** and LiBr (anion not shown).

Kimura developed more lipophilic 14-crown-4 derivatives, **7** and **8**, which incorporated dodecyl or benzyl substituents at bridgehead carbons. Ionophore **7** was incorporated in ISEs (28% wt. PVC, 70% wt. plasticizer, 1% wt. lariat ether, 1% potassium tetrakis(*p*-chlorophenyl)borate) (KT*p*ClPB) and shown to detect Li^+^-spiked blood serum from a calibration curve determined using artificial serum containing 145 mM NaCl, 4.5 mM KCl, 2.5 mM CaCl_2_, 0.8 mM MgCl_2_, 2.5 mM urea, and 4.7 mM glucose [19]. High accuracy was seen for six samples, as an average of four measurements, over the range 3 × 10^−4^ to 3 × 10^−3^ mol dm^−3^. Subsequently, Sawada et al. used **7** to detect Li^+^ in artificial serum using a flow injection system [20]. The method was robust over the range from 2 × 10^−4^ to 2 × 10^−3^ mol dm^−3^ in the presence of 0.12 to 0.16 mol dm^−3^ Na^+^, which reflected the concentration ranges of both cations in serum. Introducing a phosphate substituent, as in **8**, improved recognition for Li^+^ over K^+^ to give Li^+^:K^+^ selectivities of **7** and **8** were 229:1 and 182:1, respectively [21]. Interference by Na^+^ was an issue as the Li^+^:Na^+^ selectivities of **7** and **8** were 182:1 and 288:1, respectively. Chelating agent **9** contained six oxygen atoms as potential binding sites for Li^+^ which, as can be seen from Scheme 4, are all involved in binding the cation in a distorted octahedral environment [22]. In contrast to the complex with **5**, the anion was not coordinated, as the lipophilic substituents and oxygen atoms combined to give complete encapsulation.

**Scheme 5:**
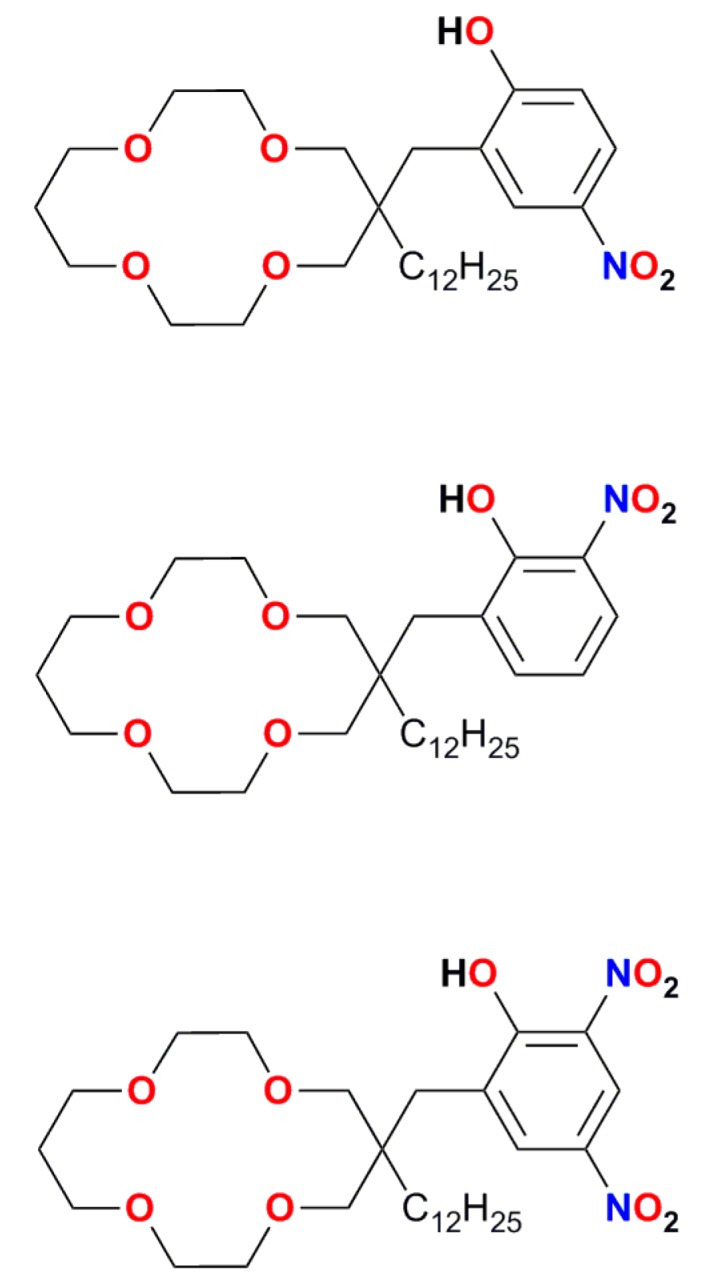
Kimura’s chromogenic 14-crown-4 ionophores: (from top) **10**, **11** and **12**.

Kimura explored the potential of 14-crown-4 to give a colorimetric response to Li^+^ binding through the introduction of nitrophenol groups in compounds **10** to **12** (Scheme 5) [23]. These ‘lariat ethers’ also incorporated a phenolic substituent which could be deprotonated to offer a fifth oxygen donor atom to coordinate to the Li^+^ cation. All showed a significant response to Li^+^, which was extracted from aqueous tetramethylammonium hydroxide into 1,2-dichloroethane with **10** exhibited an extraction equilibrium constant of 240 for Li^+^ over Na^+^, with a concomitant pale yellow to deep yellow colour change. 

**Scheme 6:**
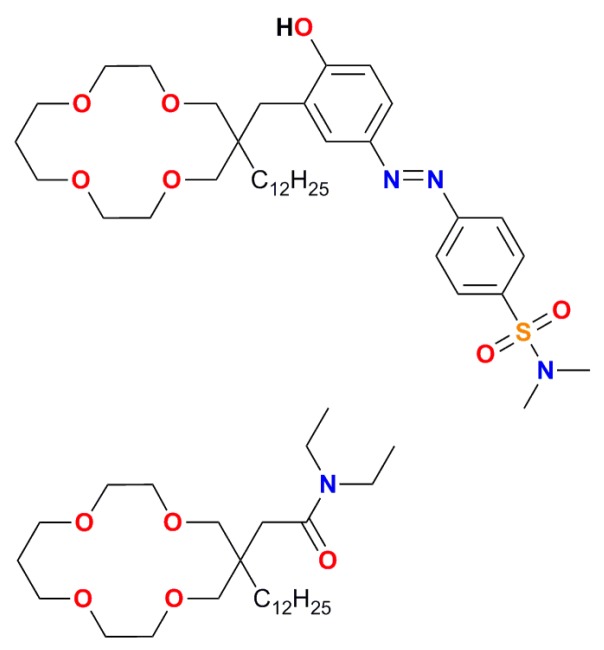
14-Crown-4 derivatives, from top, **13** and **14**.

Two other derivatives from the same group deserve mention. Diaza **13** resulted in exclusive Li^+^ extraction with a yellow to orange change in colour [23], whereas *N*,*N*-diethylacetamide-substituted **14** was the most selective for Li^+^ over Na^+^ and K^+^, out of sixteen 14-crown-4 derivatives tested (Scheme 6) [21].

**Scheme 7:**
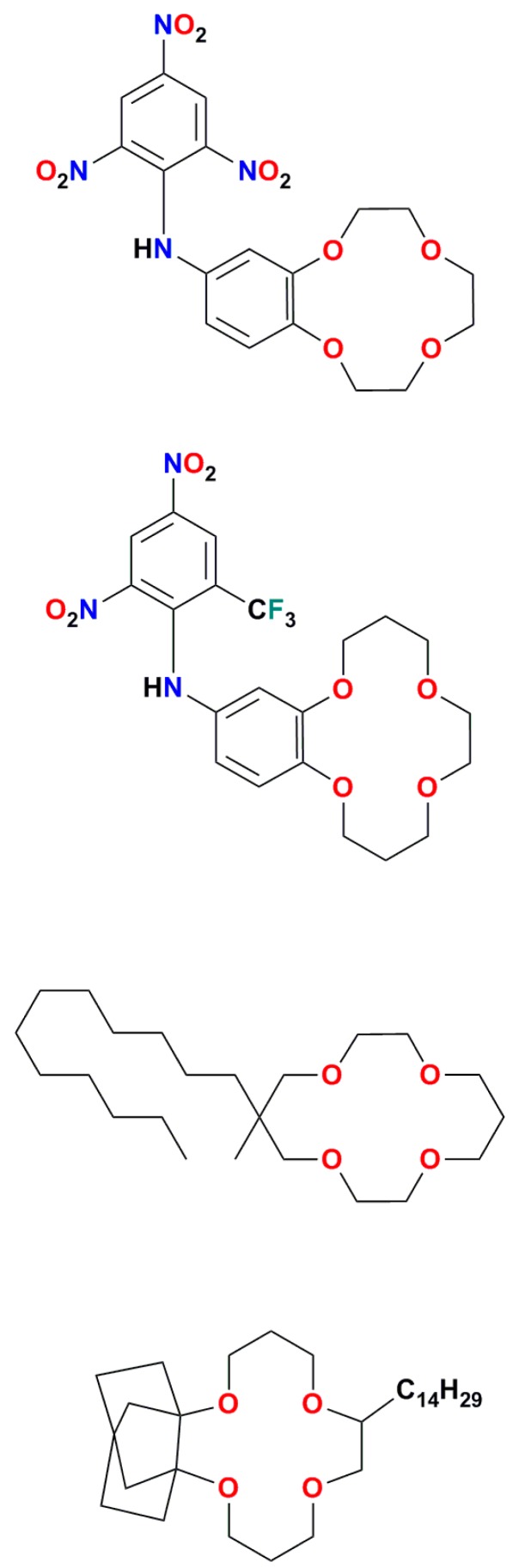
Crown ether derivatives: (from top) 4-picrylaminobenzo-12-crown-4 (**15**), 2″,4″-dinitro-6″-trifluoromethylphenyl-4′-aminobenzo-14-crown-4 (**16**), 3-dodecyl-3-methyl-14-crown-4 (**17**) and 6-tetradecyl-2,9-decahydro-14-crown-4 (**18**).

Pacey and co-workers investigated several Li^+^-specific ionophores in the early 1980s, starting with 4-picrylaminobenzo-12-crown-4 (**15**) [24] and 2″,4″-dinitro-6″-trifluoromethylphenyl- 4′-aminobenzo-14-crown-4 (**16**) [25]. Ionophore **16** was able to determine Li^+^ in treated blood serum by batch and flow injection methods, with comparable accuracy to atomic absorption (Scheme 7). 

The group led by Christian investigated the use of ionophores as modifiers in PVC membrane electrodes, and found that **4** and **17** (Scheme 7, reported previously by Kitazawa et al. [26]) gave Li^+^:Na^+^ selectivities of 140:1 and 125:1, respectively [27]. It was found that the type of plasticizer, trioctylphosphine oxide, tris(2-ethylhexyl)phosphate or *o*-nitrophenyl octyl ether, and whether or not KT*p*ClPB was added, gave significant differences in performance with Li^+^:Na^+^ selectivities ranging from 70:1 and 280:1 in electrodes made to different specifications [28,29]. 

For consistency, tris(2-ethylhexyl)phosphate was used as the plasticizer in electrodes for all but one of seventeen ionophores tested. Electrodes with this composition and the addition of **17** were able to determine the concentration of Li^+^ to within 3% error, based on atomic absorption measurements, in thirteen serum samples from patients with bipolar disorders receiving lithium-based medication [30].

The Suzuki group investigated eleven lipophilic 14-crown-4 derivatives and incorporated as modifiers in a potentiometric PVC-based Li^+^-selective electrodes [31]. Of the compounds tested, a decalin-containing derivative, **18**, gave a Li^+^:Na^+^ selectivity of over 1000 and determined concentrations of Li^+^ to within 3.4% in solutions containing 5 × 10^−4^ mol dm^−3^ Li^+^ and 0.150 mol dm^−3^ Na^+^.

**Scheme 8:**
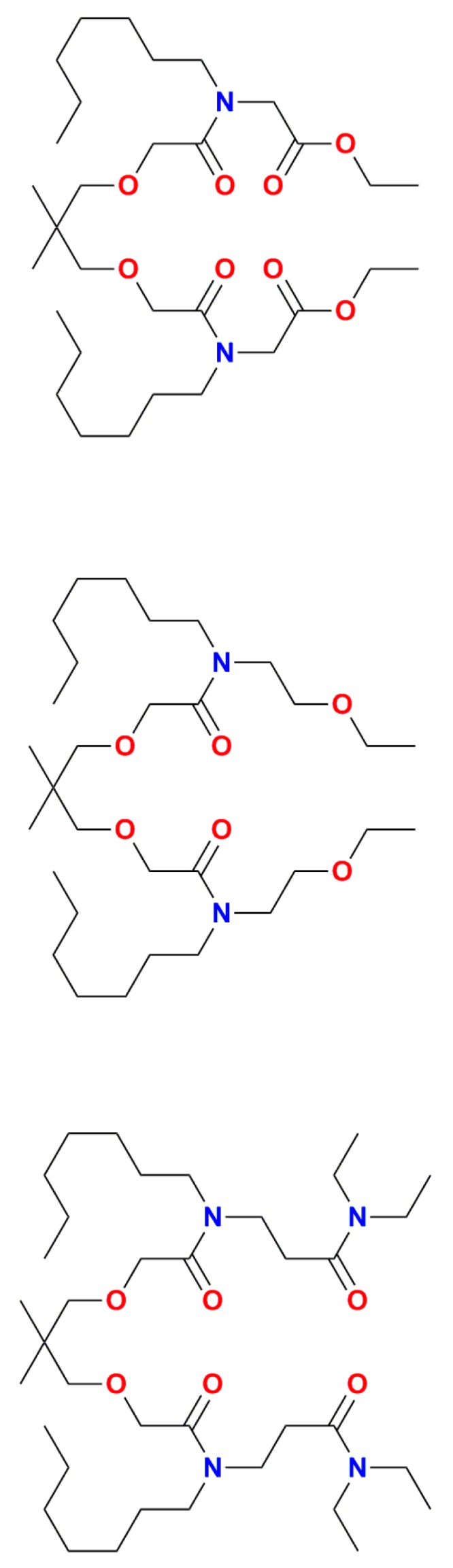
Shanzer’s acyclic ionophores: (from top) diethyl 3,13-diheptyl-8,8-dimethyl-4,12-dioxo-6,10-dioxa-3,13-diazapentadecanedioate (**19**), 2,2′-((2,2-dimethylpropane-1,3-diyl)bis(oxy))bis(*N*-(2-ethoxyethyl)-*N*-heptylacetamide) (**20**), 3,3′-(13,13-dimethyl-9,17-dioxo-11,15-dioxa-8,18-diazapentacosane-8,18-diyl)bis(*N*,*N*-diethylpropanamide) (**21**).

Recognizing that Li^+^ is often coordinated in a tetrahedral manner by acyclic lipophilic ligands incorporating oxygen atoms, Shanzer designed a group of diesters and diamides, similar to Simon’s lithium ionophore I [15], such as **19** to **21** shown in Scheme 8 [32]. In a turnaround from using these compounds as electrode modifiers to detect Li^+^, ionophore **21** was claimed to deliver Li^+^ to the brains of rats.

**Scheme 9:**
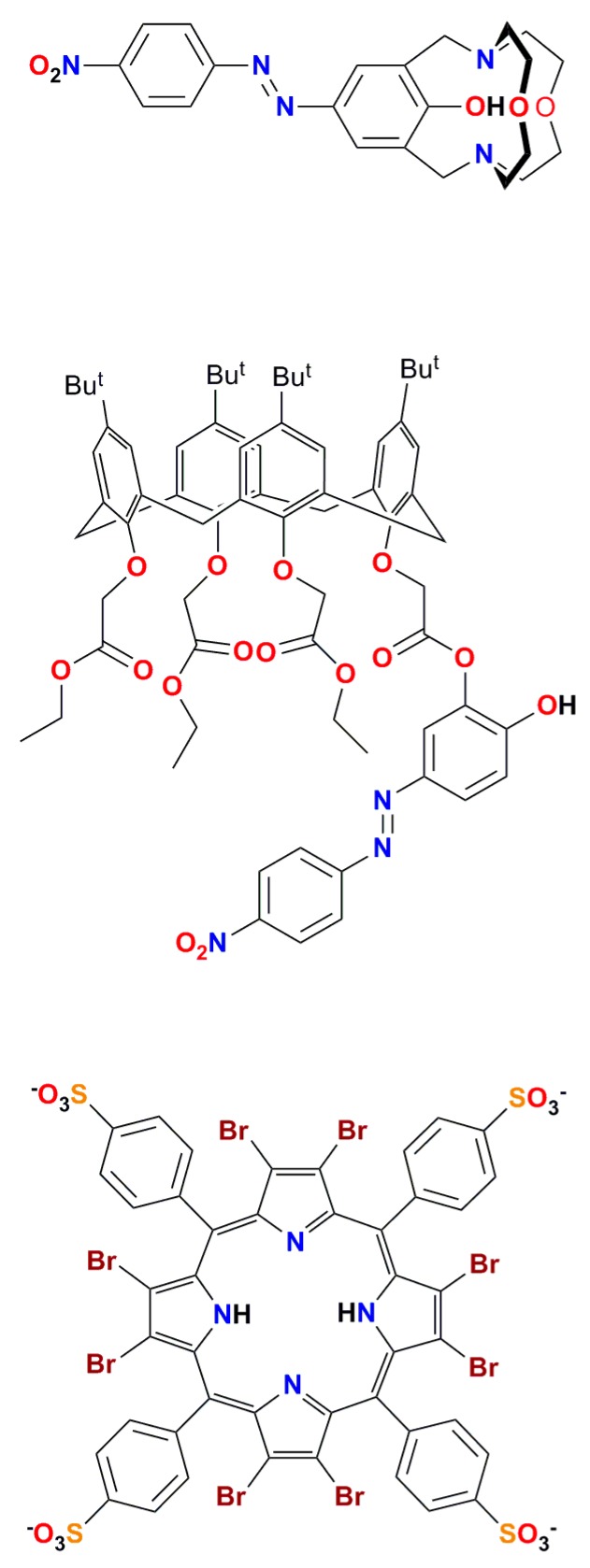
Cryptand- (**top**), calixarene- (**centre**) and porphyrin-based chromoionophores **22**, **23** and **24**.

Czech and colleagues combined the strong chelating ability of the cryptands with a [(*p* nitrophenyl)azo]phenol substituent (**22**) to produce a chromogenic Li^+^-specific ligand (Scheme 9) [33]. At pH 12 a bathochromic shift from 379 nm to 512 nm is observed for Li^+^, whereas no shift is seen for Na^+^ or K^+^. An association constant, K_a_, of 3200 M^−1^ was determined for Li^+^, but with no change in the 379 nm peak in the presence of Na^+^ or K^+^, complexation of other cations was presumed to be negligible. McCarrick et al. reported a similar response to Li^+^ in a base with the calixarene-based ionophore **23** (Scheme 9), which turned from yellow to red (380 to 520 nm) upon binding, and had a Li^+^:Na^+^ selectivity of 73.5:1 [34]. 

Tabata et al. reported that a planar, water-soluble porphyrin derivative, **24** (Scheme 9), could detect Li^+^ at concentrations between 10^−6^ and 10^−4^ mol dm^−3^ at 490 nm [35]. The method required the presence of Mg(EDTA)^2−^ in the buffer solution, and the response was unaffected by 13 common alkali, alkaline earth and transition metal cations. The chromoionophore was able to detect Li^+^ in spiked blood serum up to 10^−5^ mol dm^−3^ and in seawater to 2 × 10^−5^ mol dm^−3^, but not in hot spring water where it was present below 10^−6^ mol dm^−3^. More recently, Albero et al. incorporated ionophore **9** together with chromophore 9-[4-diethylamino-2-(octadecanoyloxy)styryl]acridine and KT*p*ClPB in a PVC membrane to measure Li^+^ in pharmaceuticals and saliva, over a 1 × 10^−4^ to 1 × 10^−2^ mol dm^−3^ concentration range [36]. Although sensitivity and linear response characteristics were good, selectivity was an issue with Li^+^:Na^+^ selectivity of 104:1, Li^+^:Ca^2+^ selectivity of 24:1 and Li^+^:Ba^2+^ selectivity of 10:1.

**Scheme 10:**
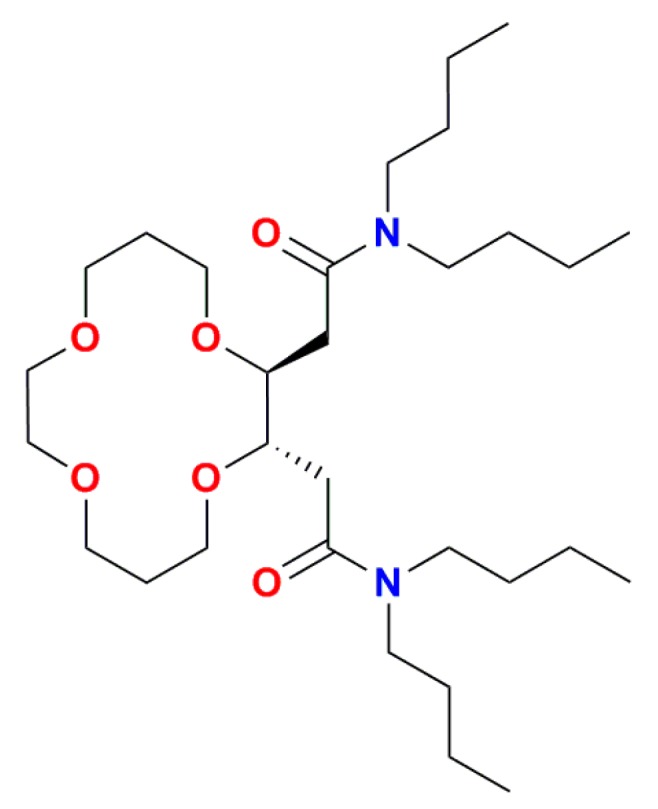
Parker’s 14-crown-4 ionophore (**24**).

In 1991, the Parker group undertook a comparative analysis of ten mono- and disubstituted 14-crown-4 ionophores using a mixture of 1.2% ionophore, 65.6% *o*-nitrophenyl octyl ether, 32.8% PVC, and 0.4% KT*p*ClPB to construct the ion-selective liquid-membrane electrodes [37]. These were tested against a commercial Li^+^-selective electrode from Corning, containing **5** as a modifier, and a Philips IS 561 electrode. Tests for Li^+^ in water and fresh blood serum found that the electrode containing **24**, shown in Scheme 10, had improved performance over both commercial electrodes.

**Scheme 11:**
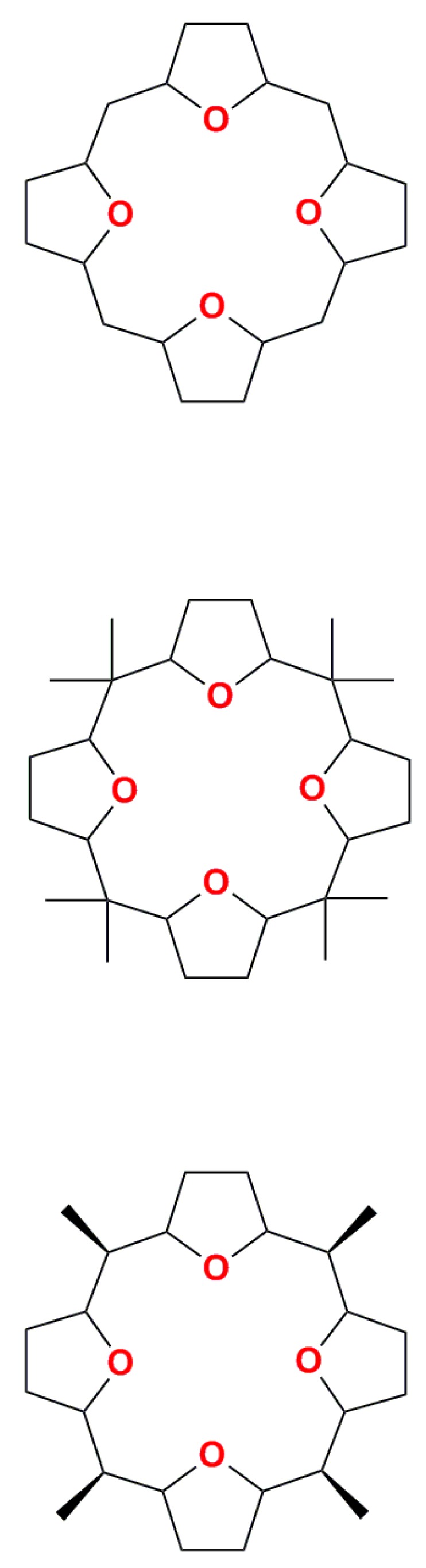
Tetrahydrofuran macrocycles: (from top) **25**, **26** and **27**.

A different design of macrocycle based on a cyclic tetrahydrofuran 16-crown-4 motif, **25**, was reported by Kobuke in 1976, where it was shown to transport Li^+^, Na^+^ and, in trace amounts, K^+^ (Scheme 11) [38]. Later work by Kim exploited the phase transport behaviour noted by Kobuke to prepare Li^+^-selective electrodes. Ion-selective liquid-membrane electrodes prepared from PVC (28% wt.), plasticiser (bis(2-ethylhexyl)adipate, dioctyladipate, *o*-nitrophenyl octyl ether, or tris(2-ethylhexyl)phosphate, 70% wt.) and ionophore with KT*p*ClPB added to a maximum 1:1 ratio with octamethyl ionophore **26** (Scheme 11), showed almost no affinity for divalent cations, and a selectivity of about 130:1 for Li^+^ over the larger monovalent cations, but only 20:1 Li^+^:NH_4_^+^ and 11:1 Li^+^:Na^+^ selectivity at best [39]. Selectivity for Li^+^ over Na^+^ was improved to 675:1 using the all-*cis* tetramethyl ionophore **27** (Scheme 11) [40]. More complex derivatives were investigated by the group in 1997, where it was demonstrated that a composition of PVC (33% wt.), *o*-nitrophenyl octyl ether (65.8% wt.), and **27** (1.2% wt.) gave a Li^+^:Na^+^ selectivity of 630:1 coupled to much improved selectivities for Li^+^ over all other cations tested [41].

**Scheme 12:**
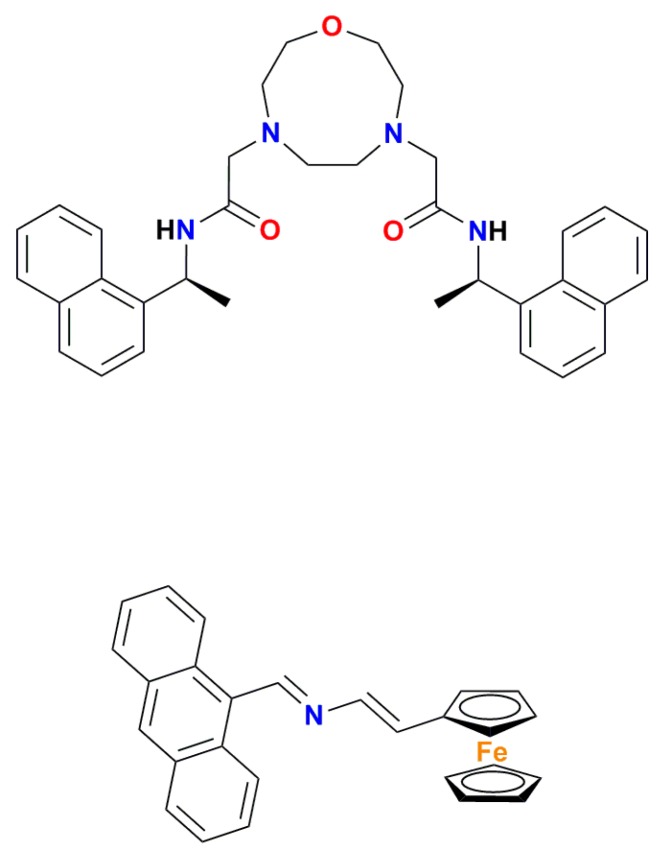
Fluorescent Li^+^ sensors **28** (**top**) and **29** (**bottom**).

Gunnlaugsson et al. coupled a diaza-9-crown-3-ether to two amidonaphthyl substituents to create a fluorescent PET (photoinduced electron transfer) sensor **28** (Scheme 12) [42]. Fluorescence quenching was observed at 337 nm in aqueous solution buffered to pH 7.4 upon the addition of LiI or LiBr; however no changes were seen for LiOAc. Using LiOAc in acetonitrile had the opposite effect; a nine-fold increase in fluorescent intensity was observed. In the same year Caballero et al. published Li^+^-selectivity data for a surprisingly simple acyclic sensor, **29** (Scheme 12) [43]. At pH 5 in 70:30 acetonitrile:water, Li^+^ exhibited a significant enhancement in its fluorescence spectrum compared to the other alkali metals.

**Scheme 13:**
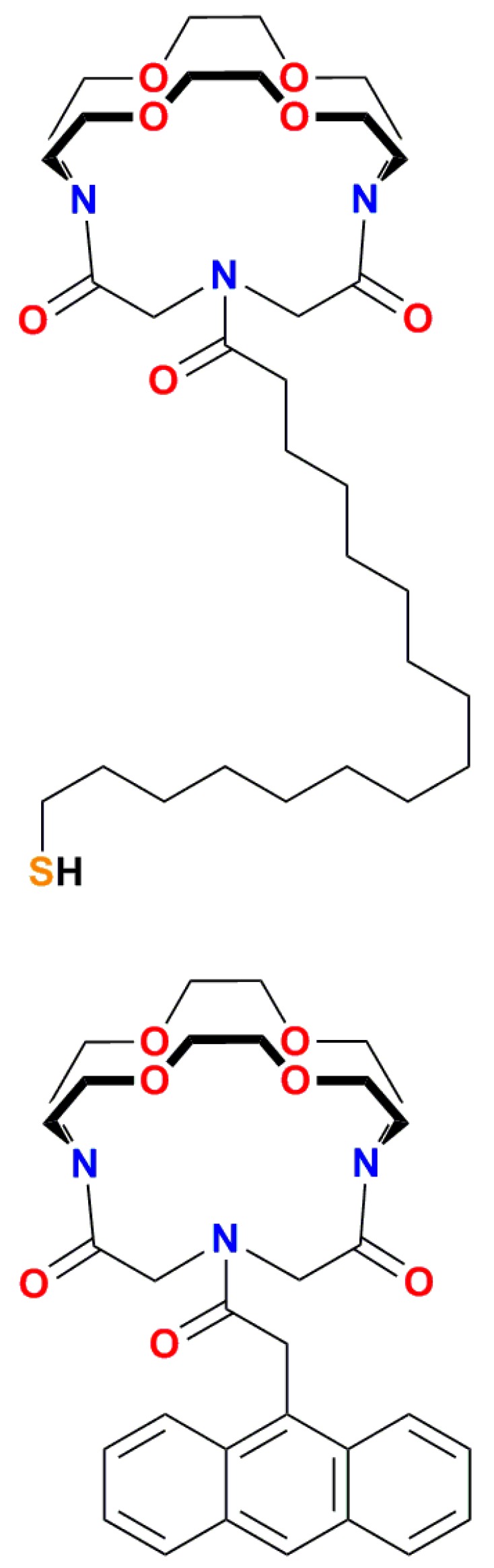
Cryptand-based sensors **30** (**top**) and **31** (**bottom**).

Wanichacheva et al. employed a cryptand attached to an alkane thiol (**30**) as the Li^+^-binding element of an electrode with a self-assembled monolayer modified surface (Scheme 13) [44]. Lehn and Sauvage showed that in aqueous solution, the stability constant, K_S_, for cryptand [2.2.1], of which **30** is a derivative, is greatest for Li^+^ over other alkali metal cations (log K_S_ for Li^+^ is 5.5, for Na^+^ it is 3.2 and for all others is less than 2.0) [45]. The response of **30** was assessed as both a SAM (self-assembled monolayer) on a gold slide, and in an ion-selective membrane comprised of dioctylphthalate (69% wt.), PVC (30% wt.) and **30** (1% wt.). The SAM-modified electrode was shown to have a Li^+^:Na^+^ selectivity of 20:1 and a Li^+^:K^+^ selectivity of 8:1, by cyclic voltammetry. This moderate selectivity was probably due to the larger cations being sandwiched between two adjacent macrocycles but this was an improvement over the ion-selective membranes with a Li^+^:Na^+^ selectivity of 6:1 and a Li^+^:K^+^ selectivity of 7:1. A fluorescent cryptand, **31**, prepared by the authors from the same molecular framework, was more promising with a Li^+^:Na^+^ selectivity of 2300:1, and a Li^+^:K^+^ selectivity of 60:1. Again, the poor selectivity for Li^+^ over K^+^ is undoubtedly due to the formation of [K·(**31**)_2_]^+^ complexes which would be more facile in solution than when the macrocycles are tethered to a surface.

**Scheme 14:**
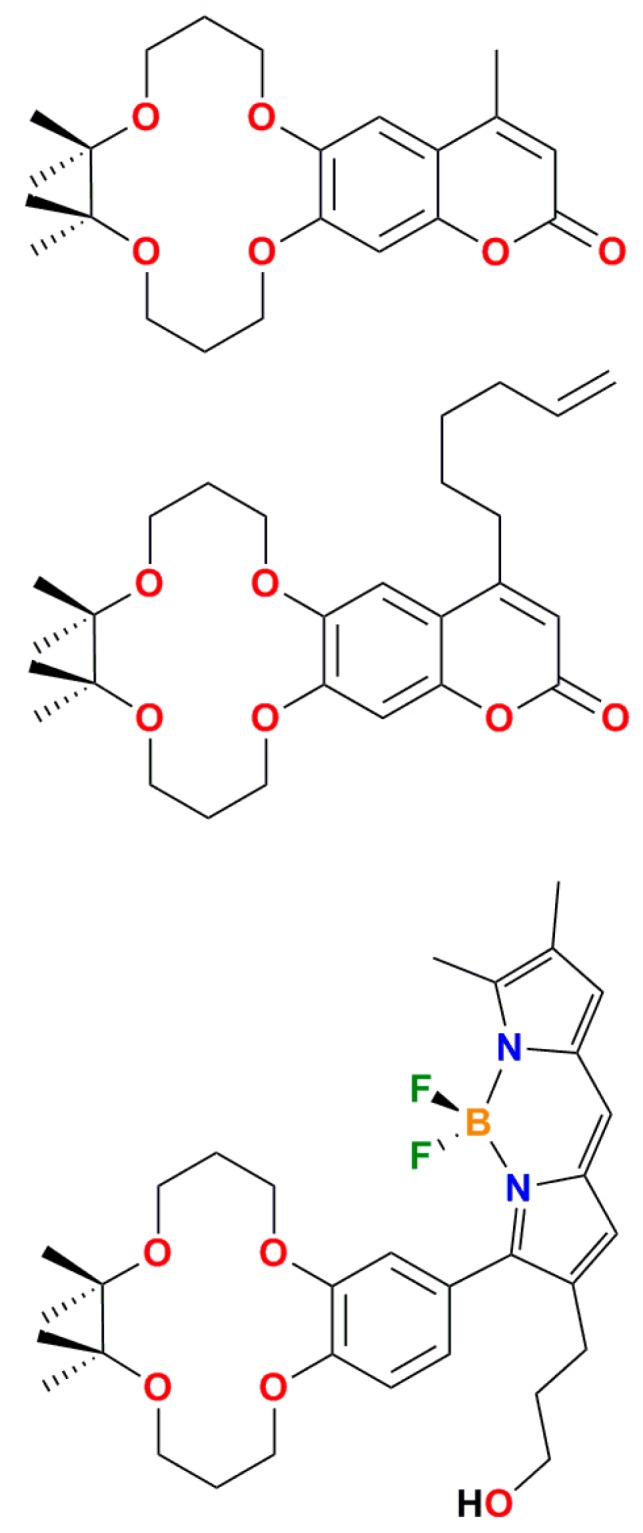
Citterio and Suzuki’s 14-crown-4 fluoroionophores: (from top) **32**, **33** and **34**.

Citterio and Suzuki developed 14-crown-4 Li^+^ fluoroionophores that incorporated a coumarin [46] or boron-dipyrromethene [47] group. In the former case, two derivatives, **32** and **33** (Scheme 14), were prepared such that molecular and polymeric systems could be studied. Studies in aqueous solution found **32** to have a Li^+^:Na^+^ selectivity of 250:1 with a working range from below 1 × 10^−4^ mol dm^−3^ to above 1 × 10^−3^ mol dm^−3^. An optode membrane for continuous monitoring in water, prepared from **33** in a matrix of *N*-vinyl-2-pyrrolidone, 2-hydroxyethyl methacrylate and dimethylacrylamide with poly(ethylene glycol) dimethacrylate (*n* = 16) as the crosslinker, gave a response time of 60 s. It was reversible, however, its Li^+^ binding strength was almost an order of magnitude larger than its non-polymerized analogue. The group’s Li^+^-selective sensor, **34** (Scheme 14), was incorporated into a glass optode. Attachment of **34** was though urethane bond formation between its terminal hydroxyl function and an isocyanate-modified glass surface. In an improvement over **33**, the material’s response was found to be reversible between 1 × 10^−4^ mol dm^−3^ to above 0.1 mol dm^−3^, and no interference was reported for Na^+^, K^+^, Mg^2+^ or Ca^2+^ up to 1 × 10^−1^ mol dm^−3^.

**Scheme 15:**
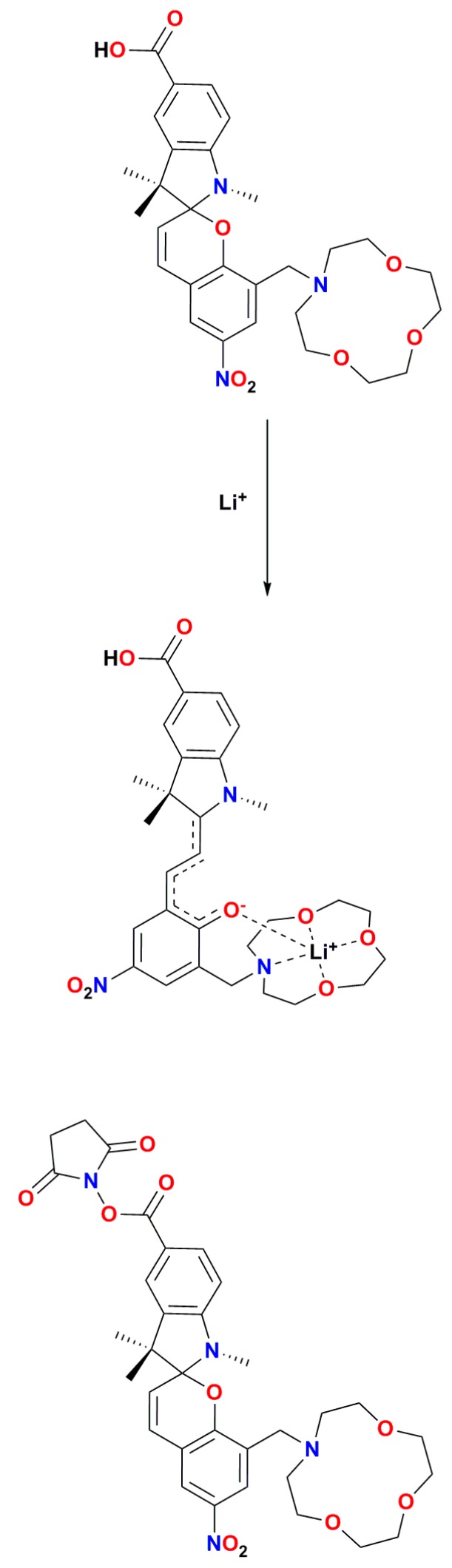
Chromoionophore **35**, illustrating its mode of action, and **36** prepared to attach to optical fibres.

Abell combined the nitrophenol chromogenic moiety and crown ether receptor first reported by Pacey [24] and introduced a spiropyran group which cleaves to bind Li^+^ in preference to other metal cations [48,49]. Investigation of chromoionophore **35** (Scheme 15) showed that exposure to ultra-violet (UV) light at 365 nm in the presence of Li^+^ generated increases in fluorescence which could be reversed with white light. Reaction with *N*-hydroxysuccinamide gave derivative **36** (Scheme 15), which could react with microstructured optical fibres treated with (3-aminopropyl)triethoxysilane. Photoswitching between UV light (365 nm for 7 min) and white light (for 12 min) demonstrated that the sensor was reversible as free Li^+^ could be washed out during the ‘off’ cycle. Furthermore, multiple ‘on’ and ‘off’ cycles could be achieved with very little attenuation of output intensity. 

**Scheme 16:**
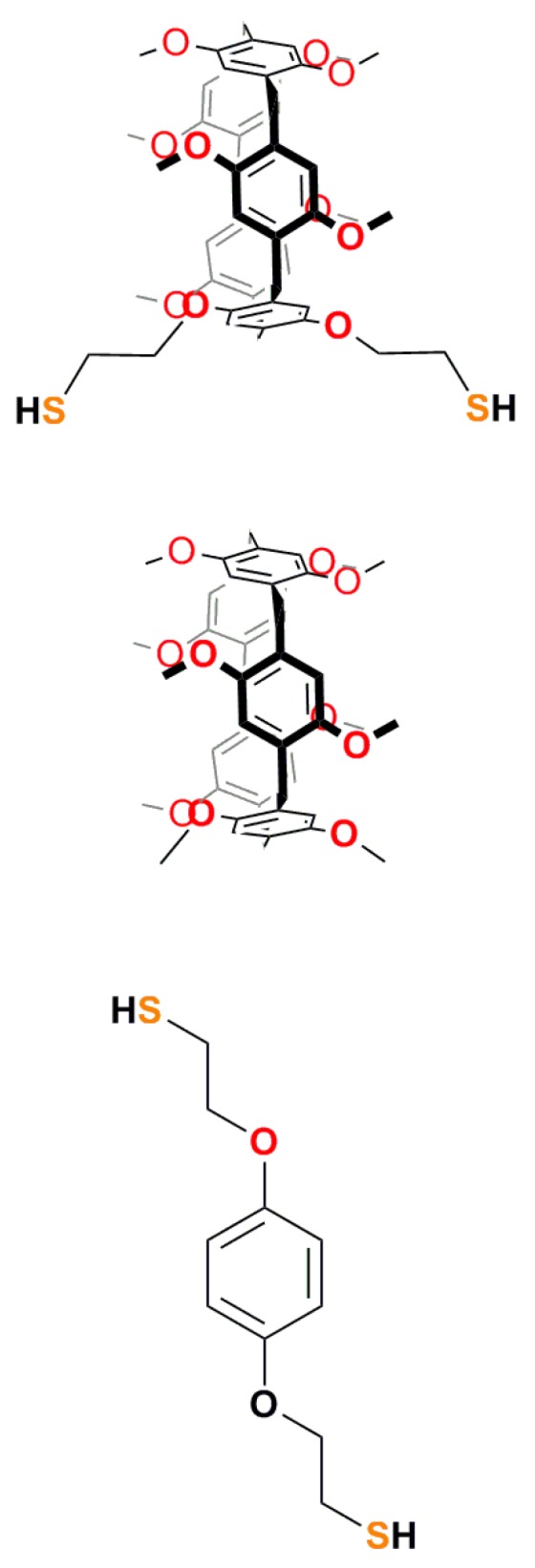
Pillar [5] arenes **37** and **38**, and monomer **39**.

**Scheme 17:**
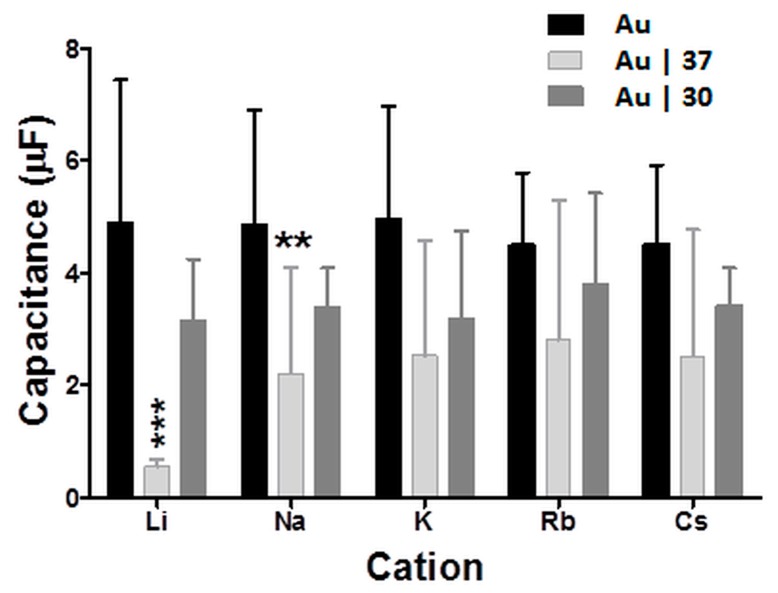
Changes observed in the capacitance signal on gold electrodes and gold electrodes coated with **37** or **39** attached (** *p* < 0.05 and *** *p* < 0.001 vs. gold electrode).

Kothur et al. attached pillar [5] arene **37** (Scheme 16) to gold surfaces though thiol links and used cyclic voltammetry to characterise the capacitive signal on 1 × 10^−2^ mol dm^−3^ alkali metal salt solutions [50]. In previous work, unmodified pillar [5] arene **38** (Scheme 16) was incorporated in an epoxy-carbon paste electrode and proved to be selective for Na^+^ [51]. This highlights the need for flexibility by surface-attached **37** which is able to adopt a conformation that preferentially bound Li^+^ over other alkali metals (Scheme 17). No selectivity was seen for monomer **39** (Scheme 16), an indication that it was the presence of the macrocycle-cation interactions and not cation-π interactions that caused selectivity.

**Scheme 18:**
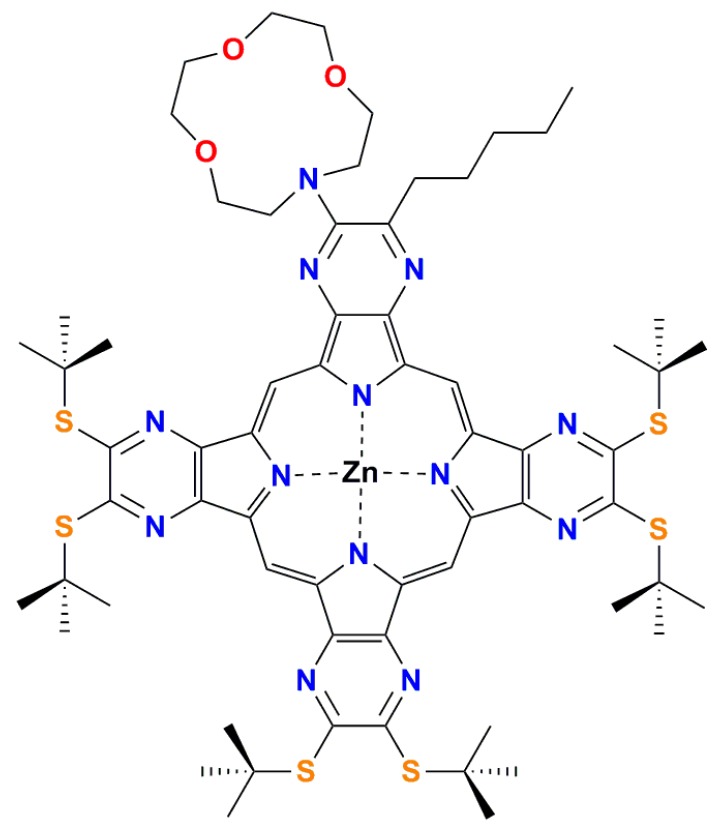
Azaphthalocyanine sensor **40**.

Lochman et al. prepared a series of crown ether-appended rigid, planar fluorescent azaphthalocyanines and demonstrated the expected selectivity based on the fit of the cation to the crown [52]. The zinc complex of azaphthalocyanine **40** (Scheme 18) gave a significant increase in fluorescence when LiClO_4_ was added. Dissociation constants, calculated from fluorescence quantum yields of the zinc azaphthalocyanines and their complexes, gave 6.4 × 10^−2^ mol dm^−3^ for Zn·**40**·Li^+^ and 1.5 × 10^−2^ mol dm^−3^ for Zn·**40**·Na^+^, with all other cations having values below 1 × 10^−2^ mol dm^−3^.

These developments in synthetic chemistry and electrode composition have had an impact on the selectivity for lithium; to illustrate this, the properties of a range of systems are given in Table 1 and compared with the detection range of a typical commercial instrument. Given the history of instrument development over the past 30 years, such advances in lithium complexation will undoubtedly lead to new and improved lithium ion sensors. 

## 4. Conclusions

The need to detect and monitor lithium both in the environment and in clinical settings has led to several approaches in sensing this element. Its emission spectrum can be used to determine its concentration, as can its X-ray fluorescence spectrum, albeit indirectly. Conductive polymers and lithium bronzes have been investigated, but ionophore-based sensors, whether colorimetric, fluorescent or electrochemical, clearly have the greatest potential for specificity and selectivity. Examples described include solution phase and surface-bound ionophores that make detection based on Li^+^-ionophore affinity possible in numerous ways, from in-line clinical monitoring to environmental sampling. As the need to accurately determine Li^+^ concentrations grows, so will the ingenuity of those methods designed for the detection of Li^+^. 

## Figures and Tables

**Table 1:** Selectivity data for selected Li^+^-responsive systems.

		Selectivity for Li^+^ Over	
Detector	Range (mM)	Na^+^	K^+^
Commercial ^a^	0.2–5	-	-
**3**	-	>100	>100
**5**	-	≈80	-
**7**	0.3–3	182	229
**8**	0.2–2	228	182
**9**	0.1–10	104	-
**27**	0.7–1.5	630	39,800
**32**	0.1– > 1	250	-

^a^ Medica Easylyte Analyzer (Medica Corporation, Bedford, MA, USA); overall sensitivity 0.01 mM for Li^+^.
